# Proteomic and Metabolomic Analyses of Right Ventricular Failure due to Pulmonary Arterial Hypertension

**DOI:** 10.3389/fmolb.2022.834179

**Published:** 2022-07-05

**Authors:** Xiaohan Qin, Chuxiang Lei, Li Yan, Haidan Sun, Xiaoyan Liu, Zhengguang Guo, Wei Sun, Xiaoxiao Guo, Quan Fang

**Affiliations:** ^1^ Department of Cardiology, Peking Union Medical College Hospital, Chinese Academy of Medical Science and Peking Union Medical College, Beijing, China; ^2^ Department of Pathophysiology, Institute of Basic Medical Sciences, Chinese Academy of Medical Sciences, School of Basic Medicine, Peking Union Medical College, Beijing, China; ^3^ Core Facility of Instrument, Institute of Basic Medical Sciences, Chinese Academy of Medical Sciences, School of Basic Medicine, Peking Union Medical College, Beijing, China

**Keywords:** proteome, metabolome, pulmonary arterial hypertension, right ventricular failure, ferroptosis

## Abstract

Right ventricular failure (RVF) is the independent and strongest predictor of mortality in pulmonary arterial hypertension (PAH), but, at present, there are no preventive and therapeutic strategies directly targeting the failing right ventricle (RV). The underlying mechanism of RV hypertrophy (RVH) and dysfunction needs to be explored in depth. In this study, we used myocardial proteomics combined with metabolomics to elucidate potential pathophysiological changes of RV remodeling in a monocrotaline (MCT)-induced PAH rat model. The proteins and metabolites extracted from the RV myocardium were identified using label-free liquid chromatography–tandem mass spectrometry (LC-MS/MS). The bioinformatic analysis indicated that elevated intracellular Ca^2+^ concentrations and inflammation may contribute to myocardial proliferation and contraction, which may be beneficial for maintaining the compensated state of the RV. In the RVF stage, ferroptosis, mitochondrial metabolic shift, and insulin resistance are significantly involved. Dysregulated iron homeostasis, glutathione metabolism, and lipid peroxidation related to ferroptosis may contribute to RV decompensation. In conclusion, we depicted a proteomic and metabolomic profile of the RV myocardium during the progression of MCT-induced PAH, and also provided the insights for potential therapeutic targets facilitating the retardation or reversal of RV dysfunction in PAH.

## Introduction

Right ventricular failure (RVF) is a complex clinical syndrome with high morbidity and mortality, and is often secondary to various cardiovascular diseases, such as pulmonary arterial hypertension (PAH), coronary artery disease, congenital heart disease, and arrythmia (Haddad et al., 2008). PAH is a rare and lethal cardiovascular disorder characterized by vasoconstriction, fibrosis, excessive proliferation, and apoptosis resistance in the pulmonary vasculature (Ryan et al., 2015). As a consequence of sustained elevation of the right ventricle (RV) afterload, RVF progression from adaptive right ventricular hypertrophy (RVH) is a principal cause of death in the end-stage of PAH, and its prognostic value has been widely considered (Hinderliter et al., 1997; Konstam et al., 2018). Some RV structure and function indices have been demonstrated to be independent predictors of mortality and treatment failure in PAH (Bustamante-Labarta et al., 2002; van Wolferen et al., 2007). In contrast to the situation for left ventricular failure, there are currently few available therapies specific to the right heart to prevent or alleviate the progression of RVF (Humbert et al., 2014).

RV adaptation and remodeling in PAH are joint results of multiple factors, including neurohormonal activation, myocardial metabolic changes, perfusion impairment, inflammation, and genetic and epigenetic dysregulations (Vonk-Noordegraaf et al., 2013). Warburg metabolism, which involves a shift from oxidative phosphorylation, disturbed glucose, and fatty acid oxidation (FAO) to increased glycolysis, plays a key role in PAH and maladaptive remodeling of the RV (Cassady and Ramani, 2020). With the activation of the autonomic nervous system and the renin–angiotensin–aldosterone system, RV compensation maintains cardiac systolic function but finally leads to dysfunction (Vaillancourt et al., 2017). Studies have also proposed that inflammation may be involved in the RV pressure overload induced by thromboembolic pulmonary hypertensin (PH) or pulmonary embolism (Watts et al., 2006; von Haehling et al., 2010). Although the pathogenesis underlying PAH has been uncovered to some extent, the key mechanism for the deterioration of RV function in PAH and their roles as treatment targets are not clearly understood.

With the development of high-throughput omics technologies, proteomics and metabolomics have been used for PAH. Faber et al. (Faber et al., 2005) first studied the proteomic alterations in hypertrophied rat RVs in the face of pressure overload and found an energy supply shift from fatty acids to glucose. The same team further found increases of stress chaperones six weeks after pulmonary artery banding (PAB), while increase in antioxidant proteins was observed 12 weeks after PAB (Faber et al., 2007). Sheikh et al. characterized the protein changes in the RV using a PAB-induced neonatal piglet model in the hypertrophied stage with mild systolic impairment and found increased structural proteins like calsarcin-1 and vinculin (Sheikh et al., 2009). Hołda et al. (Hołda et al., 2020) revealed upregulated expression of proteins associated with FAO in early PAH, while they revealed intensified fibrosis and glycolytic processes in the end-stage PAH. Zheng et al. (Zheng et al., 2018) found five metabolites in the urea cycle altered in the plasma of monocrotaline (MCT)-treated PAH rats, indicating the urea cycle disruption in the compensated stage.

Previous studies provided the omics pathogenesis mechanism of PAH. However, the whole profiles of protein and metabolite alterations during RV remodeling until failure in PAH are still unclear. Thus far, there have been relatively few studies targeting RVF, and the in-depth mechanism of RV remodeling needs further excavation. The mechanism and treatment targets underlying RV remodeling until RVF have recently been popular topics and difficult points of research.

**FIGURE 1:**
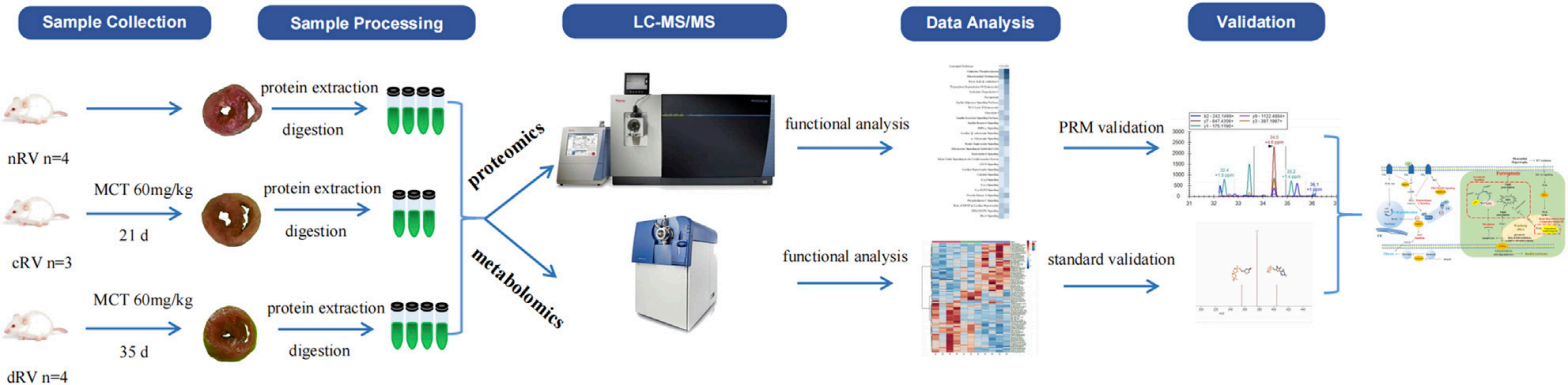
A schematic workflow of the proteomic and metabolomic analysis of the myocardium in the monocrotaline (MCT)-induced rat model of right ventricular failure.

Therefore, we tried to present a study aiming to discover protein and metabolite alterations that occur along with the deterioration of RV function, especially in the RVF stage. In this study, we analyzed the underlying mechanisms of RV remodeling and dysfunction in a commonly used MCT-induced model of PAH using the untargeted proteome- and metabolome-based high-throughput detection techniques. The results were then validated using the parallel reaction monitoring (PRM) analysis and standard substances. Significant changes in the levels of several proteins and metabolites were identified, thus providing clues for potential physiological and pathological mechanisms that may contribute to the development of updated treatments targeting RV function in PAH. An overview of the study workflow is shown in Figure 1.

## Materials and Methods

The data that support the findings of this study are available from the corresponding author upon reasonable request.

### Reagents and Instruments

MCT was purchased from Sigma–Aldrich. Sprague–Dawley rats were purchased from Vital River. Formic acid, HPLC-grade acetonitrile (ACN), dithiothreitol, and iodoacetamide were purchased from Sigma (St. Louis, MO, United States). Sequencing-grade trypsin was purchased from Promega (Madison, WI, United States). For the proteomic mass spectrometry (MS) analysis, a high-pH reversed-phase liquid chromatography (hp-RPLC) column (4.6 mm × 250 mm, Xbridge C18, 3 µm) and Orbitrap Fusion Lumos Tribrid (Thermo Scientific, Bremen, Germany) coupled with an EASY-nLC 1000 were used. For the metabolomic MS analysis, a TripleTOF 5600 MS (AB Sciex, Framingham, MA, United States) and an ACQUITY ultra-performance liquid chromatography (UPLC) system (Waters, Milford, MA, United States) were used.

### Animal Model of PAH-RVH/RVF

The animal model was established as previously described (Huang et al., 2021). Male Sprague–Dawley rats (250–300 g) were randomized into model/control groups. MCT (C2401) was dissolved in sterile saline containing 20% v/v ethanol. Intraperitoneal injection of 60 mg/kg MCT was performed to induce PAH, and the same amount of vehicle was administered to the control group. According to the data measured by echocardiography and catheterization *via* the right jugular vein in the pre-experiment, we identified the time points of compensated RV (cRV) and decompensated RV (dRV) at 21 and 35 days after MCT injection, respectively. At the end stage, 40 mg/kg pentobarbital sodium was injected intraperitoneally, and after proper anesthesia, the animals were euthanized by cervical dislocation before autopsy. The animal experiment was approved by the Institutional Animal Care and Use Committee of Peking Union Medical College (ACU​C-A01-2018-005).

### Cardiovascular Physiological, Gross Anatomical, and Histological Assessments

The RV systolic pressure (RVSP), RV end-diastolic pressure (RVEDP), and mean pulmonary arterial pressure (mPAP) were measured through catheterization *via* the right jugular vein and a connected pressure transducer. Echocardiography was performed with a Vevo 2100 platform and MS-250 transducer (FUJIFILM VisualSonics) to assess the cardiac systolic and diastolic function. The measured and calculated parameters included the RV free wall thickness (RVFW), end-diastolic RV internal diameter, tricuspid annular plane systolic excursion (TAPSE), pulmonary artery acceleration time (PAAT), and cardiac output (CO).

After autopsy, the RVFW was carefully dissected. The RV and left ventricle (LV) with interventricular septa were weighed separately. The extent of fibrosis was assessed according to the proportion of the blue-stained fibrous area on paraffin-embedded cross sections of RVs after Masson’s trichrome staining.

### Sample Preparation for LC-MS/MS Analysis

Two groups of RV samples (50 mg) were subjected to proteomic and metabolomic analyses——the study group (*n* = 11; normal RV (nRV), *n* = 4; cRV, *n* = 3; and dRV, *n* = 4) and the PRM validation group (*n* = 14; nRV, *n* = 4; cRV, *n* = 5; dRV, *n* = 5). Phosphate buffer solution was used to wash the tissues. Each sample was homogenized in 1.2 ml of 80% v/v methanol with five beads using a spinning blade tissue homogenizer (IKA R104, Janke & Kunkel KG.IKA-werk, Staufen, Germany) for complete lysis. Fully lysed tissues were transferred to new EP tubes and precipitated for 2 h at −20°C. Then, after centrifugation at 14,000 × *g* for 15 min, the supernatant and the precipitate were collected separately. The precipitate was adequately lysed using 1 ml of 2% m/m sodium dodecyl sulfate and the protein concentrations were estimated by spectrophotometry based on the BCA method. Equal amounts of normalized total protein were taken from each of the rats, and 300 μg of the protein from each sample was pooled. Proteins (300 μg) from each sample were processed by the filter-aided sample preparation (FASP) method. In brief, the protein samples were reduced with 20 mM of dithiothreitol at 95°C for 5 min and carboxyamidomethylated with 50 mM of iodoacetamide at room temperature in the dark for 45 min. After adding six times the volume of acetone, the samples were placed at −20°C for half an hour. The supernatant was discarded after centrifugation at 12,000 × *g* for 10 min, and the precipitate was dissolved with 200 µl of 20 mM Tris. Proteins from each sample were transferred to a 30 kDa filter (Millipore Corporation, United States) that had been rinsed three times in advance and centrifuged at 12,000 × *g* for 60 min. Then, the proteins were diluted with 200 μl of 20 mM Tris and subjected to another centrifugation at 12,000 × *g* until there was no liquid on the filtration membrane (repeated three times). Finally, the samples were digested with trypsin (Promega, United States; 1:50, enzyme: protein in 20 mM Tris) on the 30 kDa filter, microwaved on high temperature for 1 min twice, and incubated at 37°C overnight. After digestion, the peptides were collected by centrifugation and subsequently desalted on a C18 solid-phase extraction column (3 M, Empore, United States). The supernatant was dried under vacuum and was then reconstituted with 200 µl of 2% ACN for the metabolomic analysis. Myocardial metabolites solution from each sample was then transferred to 10 kDa filters (Millipore Amicon Ultra, MA, United States) that had been rinsed in advance with 200 µl of 2% v/v ACN to separate from larger molecules. After centrifugation at 12,000 × *g* until there was no liquid on the filtration membrane, the metabolites were transferred to the autosamplers. The quality control (QC) samples were pooled samples prepared by mixing aliquots of 11 representative samples across different groups and were therefore global representative of the whole sample set. The QC samples were injected every five samples throughout the analytical run to assess the stability and reproducibility of the method.

### Offline HPLC and LC-MS/MS for Proteomics

The samples were loaded onto a high-pH RPLC column (4.6 mm × 250 mm, Xbridge C18, 3 µm) with high separation efficiency in mobile phase A1 (H_2_O + NH_4_OH, pH 10). The peptides were eluted using 5–30% mobile phase B1 (90% v/v ACN, pH 10; flow rate, 1 ml/min) for 30 min and then collected at one fraction per minute. The 30 fractions were resuspended in 0.1% v/v formic acid after lyophilization for the LC-MS analysis.

An Orbitrap Fusion Lumos Tribrid coupled with an EASY-nLC 1000 was used for the MS analysis in the DDA and DIA modes. The peptides were loaded onto the RP C18 self-packing capillary LC column (75 μm × 100 mm, 3 μm) and were eluted using 5–30% mobile phase B2 (0.1% v/v formic acid, 99.9% v/v ACN; flow rate, 0.3 μl/min) for 60 min. The 30 fractions from RPLC were analyzed in the DDA mode to generate the spectral library. The full scan was acquired from *m/z* 350–1500 with a resolution of 60,000. The cycle time was set to 3 s, the automatic gain control (AGC) was set to 1 × e^6^, and the maximum injection time (IT) was set to 50 ms. The charge state screening included precursors with +2 to +5 charge states, and the dynamic exclusion duration was 30 s. MS/MS scans with a resolution of 15,000 were performed (isolation window: 1.6 Da; HCD collision energy: 32%; AGC target: 5 × e^4^ and maximum IT: 30 ms).

Each sample was then analyzed *via* the DIA method, in which the window lists were developed according to the DDA result of the mixed sample. The precursor ion number in each isolation window was equalized based on the *m/z* distribution of the mixed sample. The full scan was acquired from *m/z* 400–900 with a resolution of 120,000, followed by DIA scans with a resolution of 30,000 (HCD collision energy: 32%; AGC target: 1 × e^6^ and maximum IT: 50 ms). The raw data file can be freely downloaded at iProX (Integrated Proteome resources^1,2^).

1 http://www.iprox.org
2 https://www.iprox.cn/page/SSV024.html;url=16464520853472B5Q, password: bIUK.

### Offline HPLC and LC-MS/MS for Metabolomics

A Triple TOF 5600 mass spectrometer (AB Sciex, Framingham, MA, United States) was used for the metabolomic analysis. The pooled metabolite mixture or metabolite standard was first separated using a hp-RPLC column from Waters (4.6 mm × 250 mm, C18, 3 mm) at a flow rate of 0.3 ml/min with the column temperature set to 50°C. Mobile phase A was 0.1% v/v formic acid in H_2_O, and mobile phase B was ACN. Full MS acquisition was performed from *m/z* 100-1000 at a resolution of 60,000 (AGC target: 1 × e^6^, maximum IT: 100 ms). UPLC-MS/MS analyses were performed at a resolution of 15,000 (isolation window: 3 *m/z*; AGC target: 5 × e^5^; maximum IT: 50 ms). The collision energy was set at 20, 40, and 60 for each target with HCD fragmentation. The samples were injected with three technical replicates to reduce the experimental bias.

### Parallel Reaction Monitoring Analysis

PRM analysis was performed on LTQ Orbitrap Fusion Lumos combined with LC column (75 μm × 100 mm). To estimate the quality of the data, the mixed sample was used as QC during the whole analysis process, before and after all the samples, and among every seven to eight samples. The eluted gradient was 5–30% mobile phase B2 (0.1% v/v formic acid, 99.9% v/v ACN; flow rate: 0.5 μl/min) for 45 min. MS data were acquired using the following parameters: PRM mode; full scans were acquired in Orbitrap at a resolution of 60,000, while MS/MS scans were acquired with 32% normalized collision energy in HCD at a resolution of 15,000, and dynamic exclusion (exclusion size list 500, exclusion duration 30 s); the isolation window was four. Schedule mode: schedule window of 7 min.

### Statistical Analysis and Data Processing

Data are presented as the mean ± standard error of mean (SEM) or median (interquartile range), determined with the Shapiro–Wilk normality test. Comparison among >2 groups was conducted with 1-way ANOVA followed by Tukey’s *post hoc* test. All statistical analyses were performed with IBM SPSS Statistics (v. 25; IBM Corp, Armonk, NY, United States), and charts were developed with GraphPad Prism (v.8.0; GraphPad Software, La Jolla, CA, United States) and SIMCA (v. 14.1; Umetrics, Sweden).

DDA data were processed using Proteome Discoverer (Thermo Scientific, Germany) software, and searched against the rat Uniprot and SwissProt databases appended with the iRT fusion protein sequence (Biognosys, Zurich, Switzerland). The search allowed two missed cleavage sites in the trypsin digestion. Cysteine carbamidomethylation was set as a fixed modification, the parent ion mass tolerances were set to 10 ppm, and the fragment ion mass tolerances were set to 0.02 Da. The applied false discovery rate (FDR) cutoff was 0.01 at the protein level. The results were imported to Spectronaut Pulsar (Biognosys, Switzerland) software to generate a library. DIA-MS data were analyzed using the Spectronaut Pulsar (Biognosys, Switzerland) with the default settings (Yu et al., 2020). All results were filtered by a Q-value cutoff of 0.01 (corresponding to an FDR of 1.0%).

For the PRM mode, Skyline software (version 19.1.0.193) was used to select the suitable *m/z* precursor ion and *m/z* fragment ion transition for the selected peptides. The peptide settings were as follows: enzyme, trypsin [KR/P]; maximum number of missed cleavages, 2; peptide length, 8–25; variable modifications, carbamidomethyl on Cys and oxidation on Met; and the maximum number of variable modifications, 3. The transition settings were as follows: precursor charges, 2 and 3; ion charges, 1 and 2; and ion types, b, y, and p. The product ions were set the third-to-the-last ions (ion match tolerance: 0.02 Da).

Metabolomic data were processed using Progenesis QI software with the “create a new experiment,” “import data,” “review alignment,” “experimental design setup,” “peak picking,” “reviewed convolution--normalization,” and “identify compounds” functions processed automatically in sequence. The adduct ion forms which included [M + H]^+^, [M + H + Na]^+^, [M + NH_4_]^+^, [M + H – H_2_O]^+^, and [M + H – 2H_2_O]^+^ were selected as positive, and [M – H – H_2_O]^–^, [M – H]^–^, and [M + Na – 2H]^–^ were selected as negative. Further compound identification was performed by searching the Human Metabolome Database (HMDB) and Metlin database.

The relationship between proteome and metabolome signature properties and clinical traits was identified by calculating the Spearman correlation, together with corresponding *p* values. The correlation was considered significant when the Benjamini–Hochberg [BH] adjusted *p*-value <0.05.

### Functional Annotation/Bioinformatic Analysis

For the ingenuity pathway analysis (IPA), differential proteins were analyzed using IPA software (Ingenuity Systems, Mountain View, CA, United States). The proteins were mapped to the IPA database and other databases in the disease, and the functional categories and in canonical pathway categories with z-score and *p*-value rankings. For metabolite annotation, all of the differential metabolites were analyzed using the MetaboAnalyst (http://www.metaboanalyst.ca/) and the HMDB (https://hmdb.ca/).

## Results

### Progressive RV Remodeling in the MCT-Induced PAH Model

**FIGURE 2:**
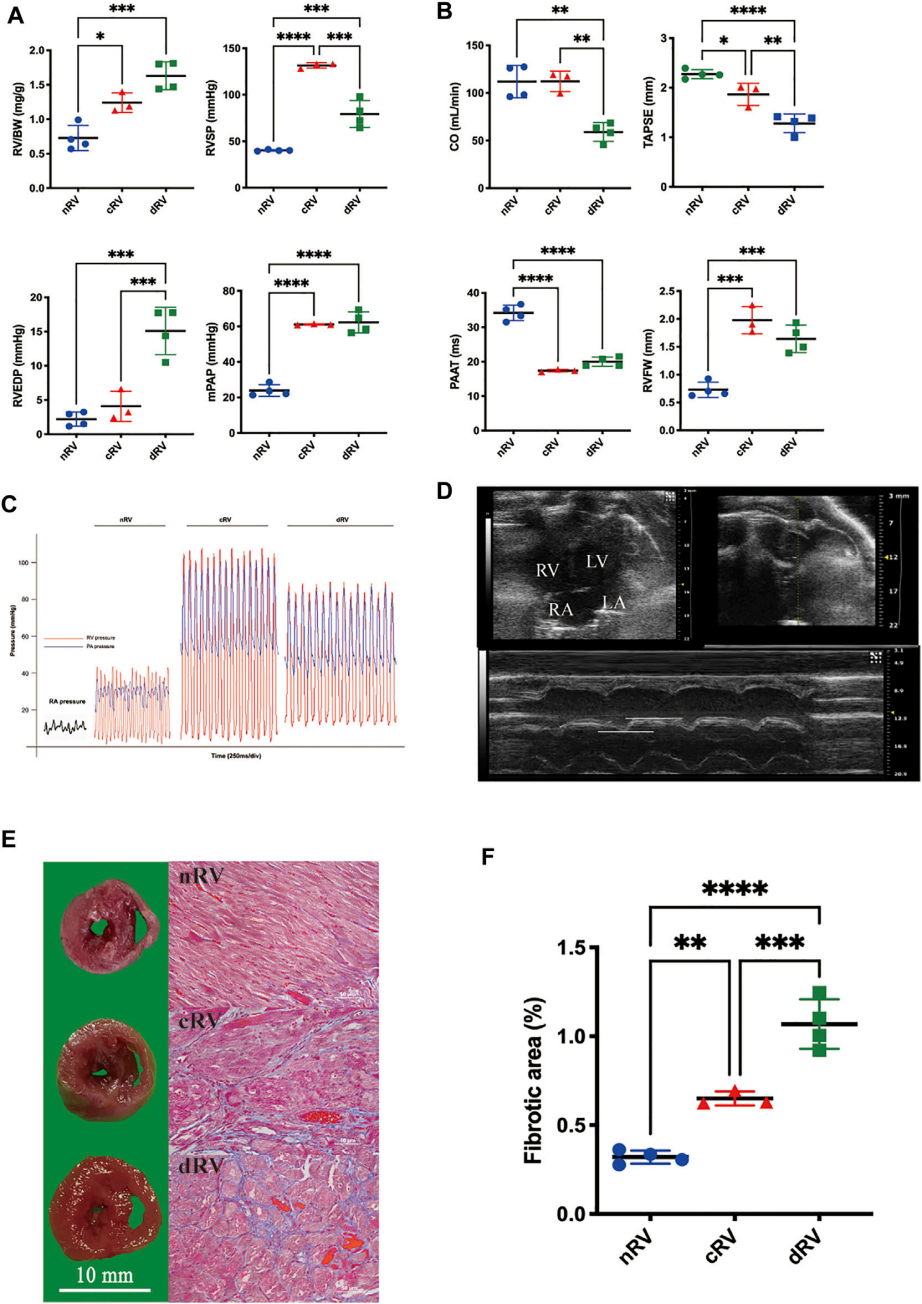
Development of the rat model of monocrotaline-induced right ventricular failure. **(A)** RV weight and hemodynamic indexes evaluated by right heart catheterization of saline- and MCT-induced rats at cRV and dRV stages. **(B)** Right ventricular function indexes evaluated by echocardiographic of saline- and MCT-induced rats at cRV and dRV stages. **(C)** Pressure waves of the rat model from right heart catheterization. **(D)** An echocardiographic image of the four-chamber view of the rat heart and the explanation of how TAPSE was measured. **(E)** Cross-sectional view of the right ventricle and the fibrotic area estimated by Masson’s trichrome stain at nRV, cRV, and dRV stages. **(F)** The column chart of the fibrotic area at different stages. nRV, normal right ventricle; cRV, compensated right ventricle; dRV, decompensated right ventricle; RV/BW, ratio of right ventricle to body weight; RVSP, right ventricular systolic pressure; RVEDP, right ventricular end-diastolic pressure; mPAP, mean pulmonary arterial pressure; CO, cardiac output; TAPSE, tricuspid annular plane systolic excursion; PAAT, pulmonary artery acceleration time; and RVFW, right ventricular free wall thickness.

In the MCT-induced PAH model, RV remodeling continues to progress, ultimately leading to RVF. Hemodynamic indices were reliable reference standards for staging (Huang et al., 2021). The nRV group presented no significant alterations in progress. In the cRV stage, rats exhibited increased afterload, shown as significantly elevated mPAP and RVSP, while in the dRV stage, rats manifested relatively lower RVSP and higher RVEDP levels (Figures 2A,C). According to echocardiography, TAPSE as an indicator of RV systolic function was decreased mildly in the cRV stage, while CO and TAPSE were decreased remarkably in the dRV stage compared to the nRV stage. The PAAT which inversely correlates with pulmonary artery pressure showed dramatic declines in both cRV and dRV stages. All these three echocardiographic indicators manifested progressive RV systolic dysfunction. The RVFW was elevated during progression, indicating progressive RVH (Figures 2B,D). Autopsy of the RV further demonstrated progressive RV remodeling with prominent interstitial fibrosis in the dRV stage and mild collagen deposition in the cRV stage (Figures 2E,F). The results of validation group rats showed the same changes (Supplementary Figure S1). In brief, the RV showed obvious hypertrophy with preserved function in the cRV stage, while in the dRV stage, the RV showed collapsed function and related manifestations, including weight loss, ascites, and pleural effusions.

### Proteomics of PAH

#### A Comprehensive Profile of the RV Proteome

To investigate the response of the RV in PAH progression, we compared the proteomic profiles of RV samples from four normal control rats not injected with MCT (nRV), three rats with cRV, and four rats with dRV after MCT injection using label-free quantitative proteomics.

In this study, pooled samples were used to establish a peptide library for the RV proteome in the DDA mode, and 4,460 proteins in total were identified. Then they were used to analyze the individual sample in the DIA mode. A total of 3,941 proteins were quantified, 3,486 proteins were found with at least two unique peptides, and 3,240 proteins were quantified in at least 50% of all 11 samples (Supplementary Table S1). To evaluate the LC-MS stability, the correlation of protein quantification in the QC samples was analyzed and the correlation coefficient of each pair of QC samples was approximately 0.98 (Supplementary Figure S2A). In addition, the technical CVs of about 93% proteins were <0.5. To reduce the technology interference, proteins with CVs >0.5 were excluded from further analysis, and a total of 3,009 proteins were retained to screen the differentially expressed proteins (DEPs).

**FIGURE 3:**
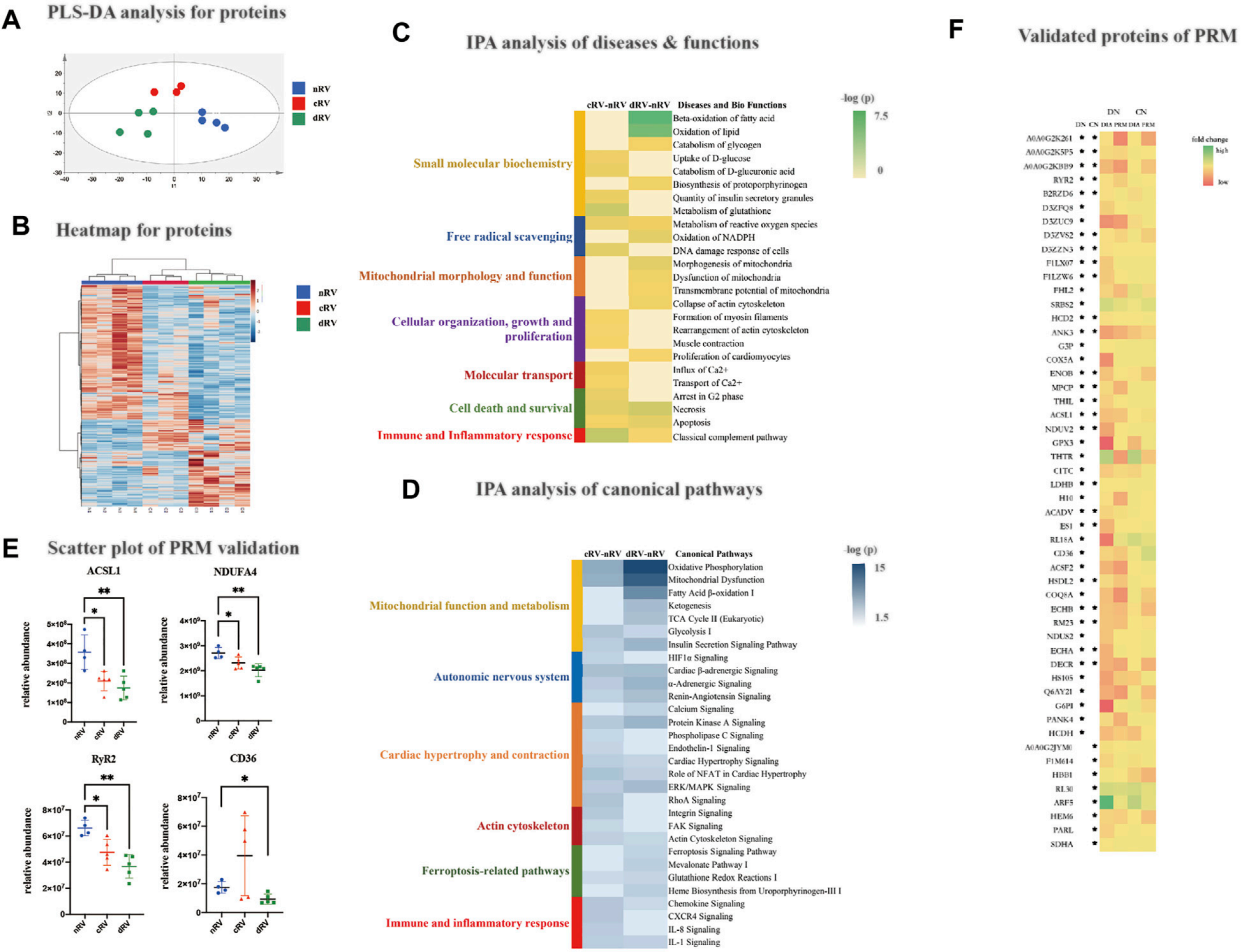
RVF proteomic analysis of the present study. **(A)** The partial least squares discriminant analysis (PLSDA) score plot based on the DIA data of the myocardial proteome from nRV rats (blue), cRV rats (red), and dRV rats (green). **(B)** The hierarchical clustering based on the quantification of differentially expressed proteins (DEPs) among different stages. **(C)** The hierarchical clustering based on the *p*-value of functions from IPA annotation. **(D)** The hierarchical clustering based on the *p*-value of canonical pathways from IPA annotation. **(E)** The scatterplots of four major DEPs’ relative abundance based on the PRM validation. **(F)** A color histogram based on the fold change in the DIA and PRM modes of the validated proteins. * The protein that was significantly altered in the corresponding group. CN, the comparison group of cRV and nRV (cRV/nRV); DN, the comparison group of dRV and nRV (dRV/nRV).

A principal component analysis was performed, and the results are shown in Supplementary Figure S2B. The separation among the three groups showed a separation trend. Then, as shown in Figure 3A, a partial least squares discriminant analysis was performed, which showed that all samples from each group gathered and the three groups separated clearly. It indicated that the proteomic characteristics of the three groups were different.

Using a fold change (FC) ratio greater/lower than 1.5/0.67 and a *p*-value less than 0.05, 296 and 480 proteins were found to be differential ones in the cRV and dRV groups, respectively, with 119 overlapping proteins (Supplementary Table S1). As shown in Figure 3B, hierarchical clustering analysis indicated that all differential proteins displayed the prominent differences among the three groups.

#### DEP Functions and Ingenuity Pathway Analysis

To further understand the biological functions, the 296 and 480 DEPs between cRV–nRV and dRV–nRV, respectively, were subjected to IPA (Supplementary Table S1). As shown in Figure 3C, the disease and biofunction analysis demonstrated that uptake and catabolism of D-glucose, rearrangement of the actin cytoskeleton, muscle contraction, influx and transport of Ca^2+^, and the classical complement pathway were significantly enriched in the cRV. Additionally, in the RV during the compensated period, we found enriched pathways related to the autonomic nervous system, cell proliferation and contraction, regulation of cellular mechanics, immunity, and inflammation (Figure 3D). Several Ca^2+^-related proteins and immune and inflammatory proteins were altered.

In the RV during the decompensated period, small-molecule biochemistry, including lipid, nucleic acid, carbohydrate, and glutathione metabolism; free radical scavenging; energy production; mitochondrial morphology and function; and organization and collapse of actin cytoskeleton were significantly enriched. Pathways related to small-molecule biochemistry and mitochondrial function, insulin sensitivity, and ferroptosis-related pathways were significantly enriched (Figures 3C,D). Proteins associated with the lipid metabolism and in regard to mitochondrial dysfunction were significantly varied. Proteins relating to iron homeostasis and the glutathione metabolism were altered. Therefore, the myocardial proteome could provide relatively comprehensive changes of the protein profiles as PAH progressed.

#### Parallel Reaction Monitoring Validation

To validate the DEPs from the study group, the PRM analysis was performed, which was a widely used targeted proteomics technology. Pooled samples were prepared by pooling 14 samples (nRV, *n* = 4; cRV, *n* = 5; and dRV, *n* = 5), and were subjected to LC-MS/MS analysis (three runs) to construct the spectrum library. Then individual samples were analyzed. Technical variation was evaluated by calculating the correlation coefficient of the mix samples (*R* ^2^ was about 0.94), and showed good LC/MS reproducibility (Supplementary Figure S2C). Skyline (version 19.1.0.193) was used to analyze the PRM results (Supplementary Table S2). In total, 32 and 44 proteins were validated in cRV and dRV, respectively, showing a consistent trend with the DIA result (Supplementary Table S2). The abundance alterations of peptides were detected, and peptides from the same protein exhibited the similar trend. As shown in Figure 3E, long-chain fatty acid-CoA ligase 1 (ACSL1), NDUFA4 relating to mitochondrial function, and ryanodine receptor 2 (RyR2) relating to calcium homeostasis were significantly altered in the cRV and dRV. Platelet glycoprotein (CD36) was significantly decreased in the dRV. A color histogram based on the fold change in the DIA and PRM modes of these 32 and 44 proteins validated in the cRV and dRV stages, respectively, comparing to the nRV stage was shown in Figure 3F.

### Metabolomics of PAH

#### A Metabolomic Profile of the RV

The metabolomic profile was analyzed with a label-free quantitative approach. A total of 862 metabolites were quantified, of which 709 metabolites were quantified in at least 50% of all 11 samples (Supplementary Table S3). The metabolites with technical CVs greater than 0.5 were excluded from further analysis to reduce the interference of technical variation, and a total of 542 RV metabolites were kept.

**FIGURE 4:**
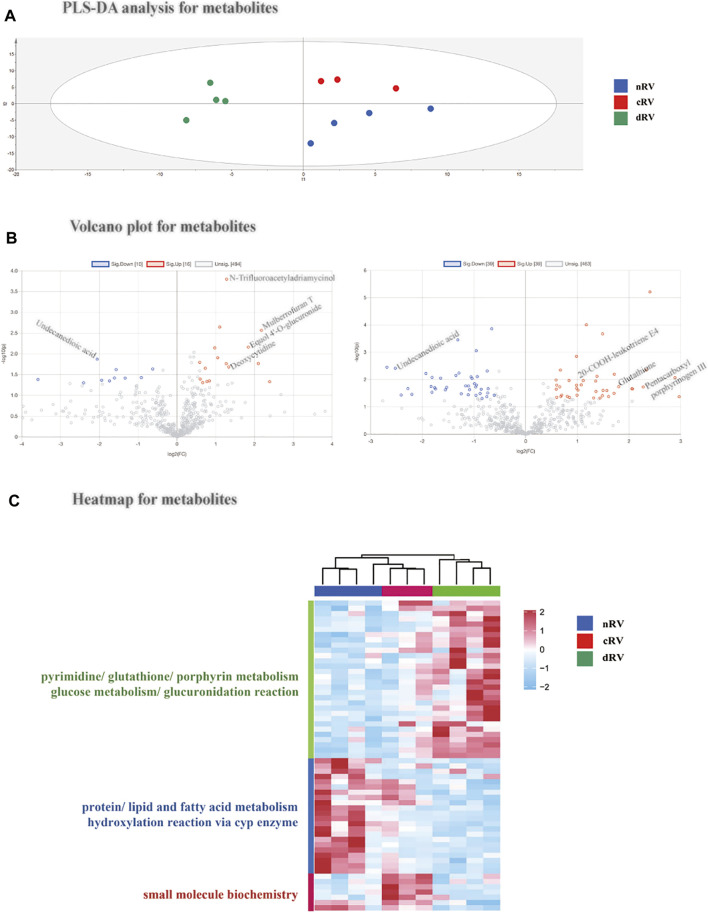
RVF metabolomic analysis of the present study. **(A)** The partial least squares discriminant analysis (PLSDA) score plot based on the quantification of the myocardial metabolome from nRV rats (blue), cRV rats (red), and dRV rats (green). **(B)** The volcano plots of the identified metabolites between cRV–nRV (left) and dRV–nRV (right). **(C)** The hierarchical clustering based on the quantification of altered metabolites among different stages and their function annotation.

Similar to the protein results, the principal component analysis and partial least squares discriminant analysis were performed using all metabolomic features, indicating that the metabolomic pattern was changed as PAH progressed (Figure 4A and Supplementary Figure S2D). Using a ratio FC greater/lower than 1.5/0.67 and a *p* value less than 0.05, 22 metabolites (14 increased and 8 decreased) were altered in cRV, and 69 metabolites (34 increased and 35 decreased) were altered in dRV (Figure 4B and Supplementary Table S3). Hierarchical clustering analysis indicated that all 59 altered metabolites in either set displayed the same changing tendency in the three groups (Figure 4C). A pie chart of the classification based on the HMDB of the altered metabolites is shown in Supplementary Figure S3.

#### Functional Analysis and Standard Validation

According to the HMDB annotation, metabolites related to glucuronidation such as Equol 4′-O-glucuronide were of higher level in the cRV stage. Metabolites with a higher level in the dRV stage include porphyrin metabolites (pentacarboxyl porphyrinogen III), pyrimidine metabolites (uridine triphosphate), and glutathione. Fatty acid metabolites, such as undecanedioic acid, were decreased in both compensated and decompensated stages, indicating FAO was downregulated in PAH. Glucuronidation metabolites, such as 5-hydroxytryptophol glucuronide, and dipeptides, such as leucyl-methionine and arginyl-asparagine, were lower in the dRV stage, indicating glucuronidation may play a potential role in PAH progression.

We further used the available metabolite standards to validate above identification, including glutathione, lumichrome, and L-phenylalanine (Supplementary Table S4).

### Correlation Analyses Between Omics Signature and RV Parameters

**FIGURE 5:**
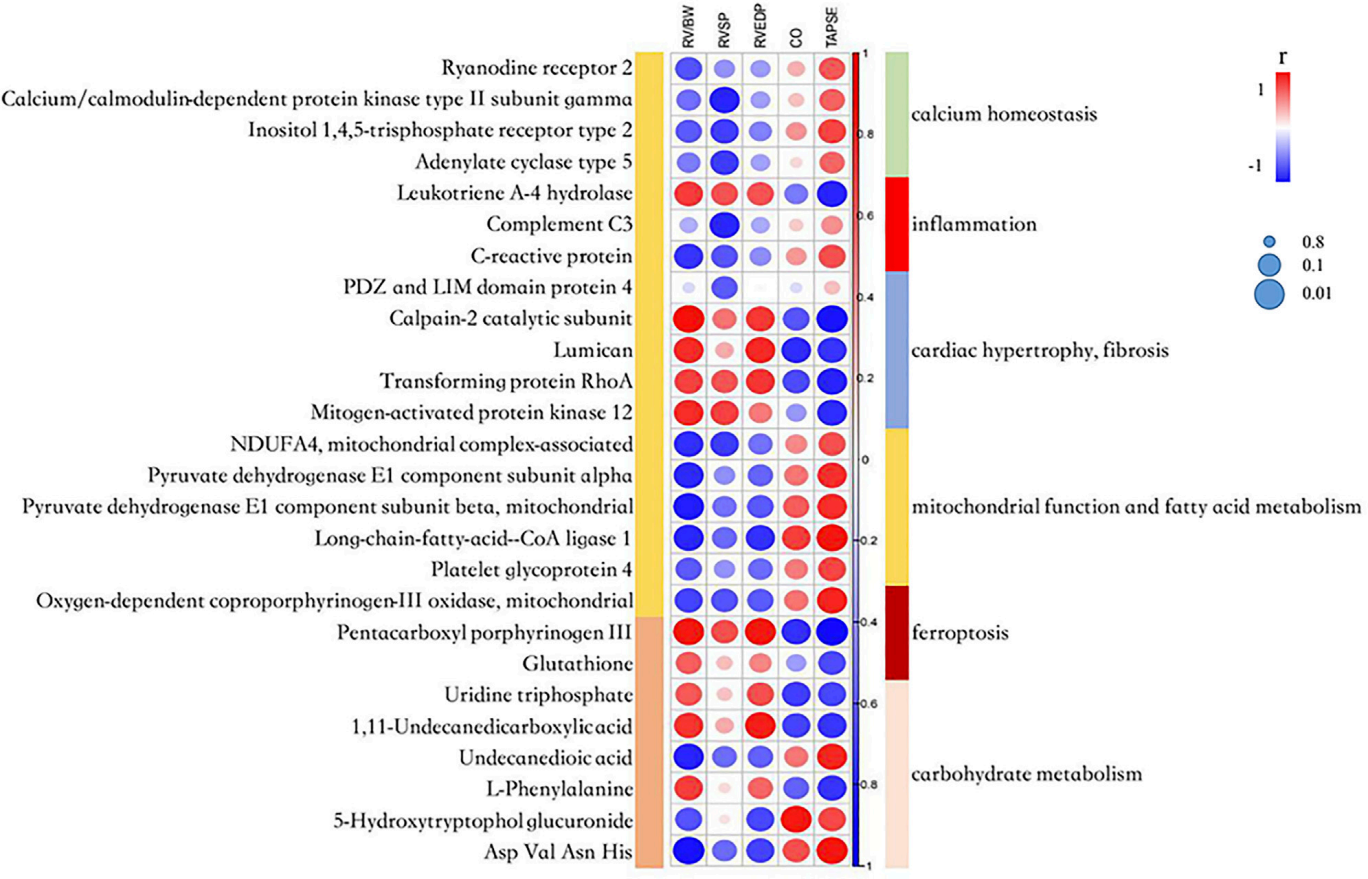
A correlation heatmap among the proteome, metabolome signatures and catherization, and echocardiography parameters. RV/BW, ratio of right ventricle weight to body weight; RVSP, right ventricular systolic pressure; RVEDP, right ventricular end-diastolic pressure; CO, cardiac output; and TAPSE, tricuspid annular plane systolic excursion. The circle’s color indicates the correlation coefficient (*r*). The size of the circle indicates the significance of the correlation according to the Benjamini–Hochberg [BH] adjusted *p*-value.

To investigate the association of clinical traits with the proteome and metabolome signatures, we made correlation analyses between proteomic and metabolomic results and clinical indicators of RV hypertrophy and dysfunction. In total, 240 DEPs and 26 altered metabolites showed a good relevance with at least one hemodynamic and echocardiographic parameter (*r* ≥ 0.7) (Figure 5 and Supplementary Table S5).

Several calcium homeostasis-related proteins had positive correlations with TAPSE and CO, and negative correlation with RV/BW and RVSP. Calpain-2 catalytic subunit and lumican related to cardiac fibrosis, and mitogen-activated protein kinase 12 (MAPK12) related to cardiac hypertrophy, showed a significantly negative correlation with CO and TAPSE, and a significantly positive correlation with RV/BW. The abovementioned results indicated that intracellular Ca^2+^ concentration and several downstream pathways might play an important role in PAH progression.

ACSL1 and two subunits of pyruvate dehydrogenase involved in the TCA cycle showed a significantly positive correlation with TAPSE and a significantly negative correlation with RV/BW, indicating that mitochondrial dysfunction and Warburg effect transition contributed to RV dysfunction. The fatty acid metabolite, undecanedioic acid, had a positive correlation with TAPSE and a negative correlation with RV/BW, which was consistent with the fact that FAO was downregulated as the RV dysfunction progressed.

The metabolite, pentacarboxyl porphyrinogen III which was involved in heme biosynthesis and affected iron concentration, had a negative correlation with TAPSE and CO, and a positive correlation with RVEDP and RV/BW. Glutathione relating to ferroptosis had a similar correlation trend. The abovementioned data further demonstrated that intervened ferroptosis may affect disease progression.

## Discussion

**FIGURE 6:**
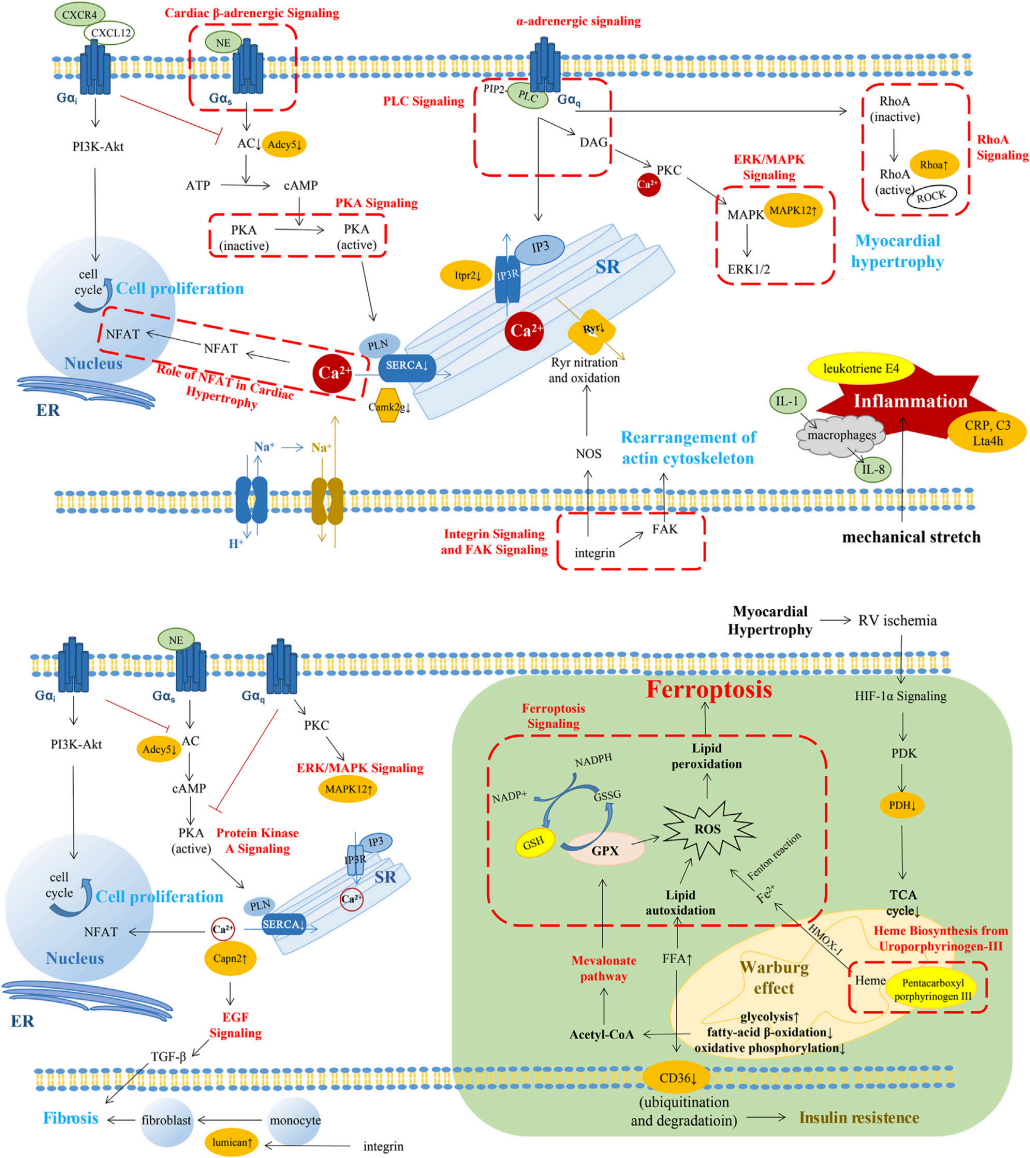
A summary pattern describing the key changes in the molecular and pathway level through RVF progression. The changes in the compensated stage of RV (above). The changes in the decompensated stage of RV (below). The changes in green background represented our novel findings. The pathways in red font or in red dashed box were the altered pathways which were the same with previous studies. Molecules with orange highlights represented the importantly altered proteins in our results. Molecules with bright yellow highlights represented the importantly altered metabolites in our results. SERCA2, sarcoplasmic reticulum (SR) Ca^2+^-ATPase; PIP2, phosphatidylinositol-4,5,-bisphosphate; IP3, inositol-1,4,5-trisphosphate; Itpr2, inositol 1,4,5-trisphosphate receptor type 2; Camk2g, calcium/calmodulin-dependent protein kinase type II subunit gamma; ADCY5, adenylate cyclase type 5; MAPK, mitogen-activated protein kinase; DAG, diacylglycerol; NOS, nitric oxide synthase; NFAT, nuclear factor of activated T cells; ROCK, the RhoA-Rho kinase; Lta4h, leukotriene A-4 hydrolase; GPX4, glutathione peroxidase 4; HIF-1α, hypoxia-inducible factor 1α; PDK, pyruvate dehydrogenase kinase; PDH, pyruvate dehydrogenase; and FFA, free fatty acid.

In this study, we analyzed the proteome and metabolome of the RV myocardium, identified DEPs, and altered metabolites in cRV and dRV compared with nRV, which deepened our understanding of changes in RV adaptation and failure in the MCT-induced rat model of PAH. It may provide clue to mechanism exploration and promote therapeutic advances. According to the functional annotation, we established a pattern describing the major extrapolated changes in the molecular and pathway level through RVF progression (Figure 6).

### Compensated Stage of RV Changes

In the early phase of RV alteration during PAH progression, compensatory hypertrophy of RV could maintain the preserved function with the increase of myocardial growth and contractility to fit the elevated afterload (Maillet et al., 2009; Swildens et al., 2010; Maron and Leopold, 2015; Vaillancourt et al., 2017; Cassady and Ramani, 2020; Maron et al., 2020; Marunouchi et al., 2021). In our results, we found pathways, such as calcium signaling, protein kinase A signaling, phospholipase C signaling, and endothelial-1 signaling, leading to the variation of Ca^2+^ concentration. A previous study has reported that stimulation of β-adrenoceptors facilitates GαS coupling and further activates adenylyl cyclase/cyclic adenosine monophosphate/protein kinase A signaling and L-type calcium channel (Maron and Leopold, 2015). Inactive protein kinase A results in decreased phosphorylation of phospholamban and lower activity of sarcoplasmic reticulum Ca^2+^-ATPase, leading to insufficient diastolic Ca^2+^ clearance in RV contributing to RV diastolic dysfunction (Rain et al., 2014; Maron and Leopold, 2015). Gαq-coupled receptors which can be promoted by the stimulation of α2-adrenoceptor can activate phospholipase C-β. It converts phosphatidylinositol-4,5,-bisphosphate into inositol-1,4,5-trisphosphate and diacylglycerol, which increase the intracellular Ca^2+^ levels and activate protein kinase C (Maron et al., 2020). Mechanical stretch and β-adrenergic signaling could induce renin–angiotensin II to activate AT1R and release endothelial-1. Endothelin receptor could initiate Na^+^-H^+^ exchange and further promote Na^+^-Ca^2+^ exchange (von Lewinski et al., 2004). In addition, mechanical stretch could activate the integrin signaling pathway and then activate neuronal nitric oxide synthase, resulting in RyR nitration and release of Ca^2+^ from sarcoplasmic reticulum (Swildens et al., 2010).

The abovementioned pathways might result in the elevation of intracellular Ca^2+^ concentration, which could activate multiple downstream pathways relating to cell growth, proliferation, and contractibility (Zarain-Herzberg et al., 2011). In this study, we found altered cardiac hypertrophy signaling and related nuclear factor of activated T cells (NFAT), ERK/MAPK signaling, and RhoA signaling. Our previous study found that diastolic Ca^2+^ leakage from the sarcoplasmic reticulum *via* oxidized Ryr2 promoted RV dysfunction in PAH (Huang et al., 2021). In cardiomyocytes, the Ca^2+^/calmodulin–calcineurin–NFAT pathway is one of the main Ca^2+^-dependent pathways, and stimulation of calcineurin-NFAT activity could promote myocardial hypertrophic growth and contribute to myocardial fibrosis (Maillet et al., 2009; Marunouchi et al., 2021). Moreover, diacylglycerol could activate protein kinase C and the downstream MAPK signaling pathway, which was involved in cardiac hypertrophy (Luo et al., 2019). Upregulated α_1_-adrenoceptor-G_q_ signaling in the failing myocardium could increase the myofibrillar Ca^2+^ sensitivity through the RhoA–Rho kinase (ROCK) pathway (Suematsu et al., 2001). Activated ROCK could further stimulate smooth muscle contraction, tissue fibrosis, and pro-inflammatory effects (Phrommintikul et al., 2008). The adaptive response of RV to pressure overload may be attributed to the aforementioned changes.

As shown in our results, inflammation may also play an important role in the cRV stage. Our proteomic results showed canonical pathways such as chemokine signaling, CXCR4 signaling, IL-8 signaling, and IL-1 signaling were significantly enriched in the CN set (cRV/nRV). In the metabolomic results, we found that 20-COOH-leukotriene E4 was elevated in the DN set (dRV/nRV). Several studies have shown that inflammation was involved in the pressure-overloaded RV, such as the participation of macrophages, T-regulatory cells, interleukin, chemokines, and leukotriene (Watts et al., 2006; von Haehling et al., 2010; Vonk-Noordegraaf et al., 2013; Sydykov et al., 2018; Mamazhakypov et al., 2021). IL-8 is produced from several cell types like macrophages and epithelial cells. The production would increase in response to TNF-α and IL-1 stimulation (Maron et al., 2020). CXCR4 is widely expressed in multiple cells and tissues including T lymphocyte, macrophages, neutrophils, lung, and heart (Hughes and Nibbs, 2018). Several studies have demonstrated that CXCR4 could promote cell proliferation in pulmonary vasculature and some tumor cells *via* PI3K/Akt and RhoA/ROCK signaling pathway (Wei et al., 2015; Mu et al., 2018; Singh et al., 2020; Wu et al., 2020). CXC-chemokine ligand 10, 12, and 16 (CXCL10, 12, and 16) were reported to be associated with right ventricular dysfunction in idiopathic PAH patients (Tao Yang et al., 2014). CXCL12, the ligand of CXCR4, was significantly increased in the RV of PH mice (Young et al., 2009). Neutralization of CXCL12 could attenuate PH in rats and even had a better beneficial effect than CXCR4 blockade (Bordenave et al., 2020). Chemokines can contribute to myocardial fibrosis through the expression of proteoglycans from cardiac fibroblasts, which have an adverse impact on RV in PAH (Waehre et al., 2012; Pilling et al., 2015).

### Decompensated Stage of RV Changes

With the progression of PAH, RV gradually proceeded into the maladaptive stage with insufficient adaptation of the capillary density and myocardial ischemia (Ruiter et al., 2013). In our results, ferroptosis signaling pathway, mevalonate pathway I, glutathione redox reaction, and heme biosynthesis were changed in dRV with the alteration of proteins such as glutathione synthetase and transferrin receptor as well as metabolite glutathione. Ferroptosis is a novel form of regulatory cell death characterized by the iron-dependent accumulation of lipid peroxides to lethal levels (Fang et al., 2019). It has been reported to link with several cardiovascular diseases such as cardiomyopathy, stroke, and ischemia/reperfusion injury (Fang et al., 2019; Fang et al., 2020). Iron homeostasis, one of the key parts of ferroptosis, was involved in pulmonary hypertension indicating iron replacement as a potential therapy for PH (Cotroneo et al., 2015). A previous study showed that inhibiting mitochondrial lipid accumulation or lipid peroxidation had protective effect on myocardium as ferroptosis was implicated in cardiomyopathy through the Nrf2/Hmox1 axis (Fang et al., 2019). The inhibition of glutathione peroxidase 4 or its cofactor glutathione could suppress the reduction of lipid peroxides, leading to intracellular accumulation of ROS (Wan Seok Yang et al., 2014). The mevalonate pathway could mediate ferroptosis by decreasing glutathione peroxidase 4 and CoQ10, an antioxidant in cells (Lei et al., 2019). The major pathways of generation of lipid peroxides in ferroptosis are iron-dependent and almost all iron is transported into cells by a transferrin-dependent manner *via* binding to the transferrin receptor.

In the cytoplasm, most of iron is transported into mitochondria for the synthesis of heme (Wan Seok Yang et al., 2014). In the dRV stage, heme-biosynthesis was decreased with altered pentacarboxyl porphyrinogen III combined with the accumulation of fatty acid due to metabolism switch, contributing to the increase of intracellular iron concentration, which could in turn cause deterioration of mitochondrial function and dynamics (Sumneang et al., 2020). In the functional analysis of DEPs, mitochondrial morphology and function, small-molecule biochemistry including lipid, nucleic acid, and glucose metabolism was significantly altered in the dRV compared to normal controls. The metabolism alteration of the myocardium related to mitochondrial dysfunction is a remarkable characteristic of RVF. A shift from aerobic FAO and oxidative phosphorylation toward anaerobic glycolysis plays a pivotal role in maladaptive remodeling of RV (Hemnes et al., 2014; Talati et al., 2016). Long-chain fatty acids, ceramides, and triglycerides were increased in the RV, suggesting the impact of fatty acid metabolism changes and lipotoxicity in PAH (Brittain et al., 2016). This transition named the Warburg effect is mediated by hypoxia-inducible factor 1α, activating pyruvate dehydrogenase kinase, an inhibitor of mitochondrial pyruvate dehydrogenase, which is a critical point for pyruvate into the TCA cycle (Piao et al., 2010; Sutendra et al., 2014; Culley and Chan, 2018).

Relating to the lipid metabolism, CD36, a vital free fatty acid (FFA) transporter mediating the majority of FFA uptake in cardiomyopathy, also showed a decrease in dRV in our results. FFA transportation is closely related to insulin sensitivity and the expression level of CD36 is in proportion to it (Talati et al., 2016). The dysfunction of mitochondria promotes lipid accumulation and impaired FAO, and high concentration of FFA could facilitate ubiquitination of CD36, accelerating its degradation (Smith et al., 2008). Insulin resistance is highly prevalent in PAH and is associated with worse ventricular function (Brunner et al., 2014). Previous studies have demonstrated that insulin resistance in PAH is not owing to an increased secretion of insulin by the pancreas, but due to the disordered lipid metabolism which may incur inflammation *via* oxidized LDL-mediated vascular injury (Maron et al., 2020). Metformin, a drug aiming to enhance insulin sensitivity and FAO, has been reported to have the ability to improve RV function in mouse models (Brittain et al., 2019). A single-center, open-label trial has verified that metformin therapy in idiopathic/heritable PAH patients could improve the RV fractional area change (Brittain et al., 2020).

The alterations of the autonomic nervous system and downstream Ca^2+^ homeostasis in the dRV stage were deteriorated to some extent. Chronically increased adrenergic stimulation leads to downregulation of β-receptors in the RV myocardium and reduced norepinephrine storage in the dRV stage (Bristow and Quaife, 2015). In our results, canonical pathways like cardiac β-adrenergic signaling, protein kinase A signaling, and α-adrenergic signaling were inhibited in dRV compared with nRV with the downregulation of adenylate cyclase type 5. In the failing RV of PAH patients, adrenergic signaling was attenuated with decreased β1-adrenoceptor density and exceptional activity of the catalytic subunit of adenylyl cyclase (Maron et al., 2020). Myocardial fibrosis was reported to be involved in the dysfunction of RV (Rain et al., 2013). Angiotensin II may have a profibrotic effect in PAH (Sun et al., 1997; Friedberg et al., 2013). In addition, the upregulated calpain-2 catalytic subunit may promote cardiac fibrosis *via* EGF and TGF-β. Small leucine-rich proteoglycans such as lumican have been studied as mediators of cardiac fibrosis (Pilling et al., 2015; Chen et al., 2020).

## Conclusion

In conclusion, our study presented a comprehensive profile of RV proteome and metabolome which depicted the disease progression of PAH and concomitant RVF. In the compensated stage, neurohormonal activation, Ca^2+^ homeostasis, and inflammation may contribute to RVH with preserved RV function. In the decompensated stage, mitochondrial dysfunction, insulin resistance, and lipotoxicity are probably interrelated in the dRV stage, and treatment targeting cardiac myocardial metabolism may be a promising method. In addition, the specific relevance between ferroptosis and PAH as well as RV function is still awaiting for further excavation. If metabolic interventions, therapeutic effect of metformin, and ferroptosis inhibition could retard the deterioration of RV function, further validation is needed in higher evidence-based studies.

For the prospects of future research, the following aspects could further be improved. First, the sample size in this study was relatively small; more samples would be used to validate the results of this study. Second, we used the MCT-induced rat model, which was a single pathological insult model, and MCT may have some toxicity on the myocardium and other organs. Thus, the results in our study should be validated in other PAH animal models, related cell lines, and clinical samples. Third, our study focused on the changes between the compensation and decompensation stages comparing to the control stage. Whether there are other pathways that we have not discovered between the compensation and decompensation states needs further studies. Fourth, our study is a pilot omics study, and the concrete mechanism pathway of the disease needs more functional experiments grounded in basic research.

## Data Availability

The datasets presented in this study can be found in online repositories. The names of the repository/repositories and accession number(s) can be found in the article/Supplementary Material.
