# Supplementary figures and images for: Proteomic and Metabolomic Analyses of Right Ventricular Failure due to Pulmonary Arterial Hypertension
Source: Front Mol Biosci. 2022 Jul 5;9:834179. doi: 10.3389/fmolb.2022.834179 (PMC9294162; doi:10.3389/fmolb.2022.834179)

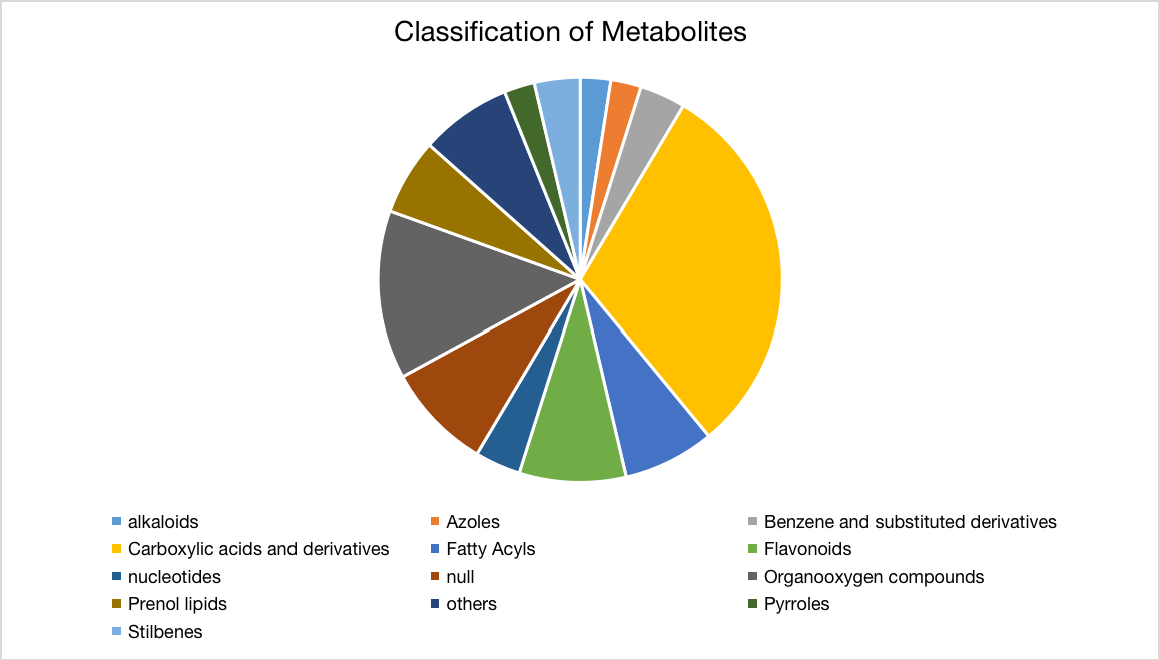

Supplement: Supplementary file 2 [file Image3.JPEG]

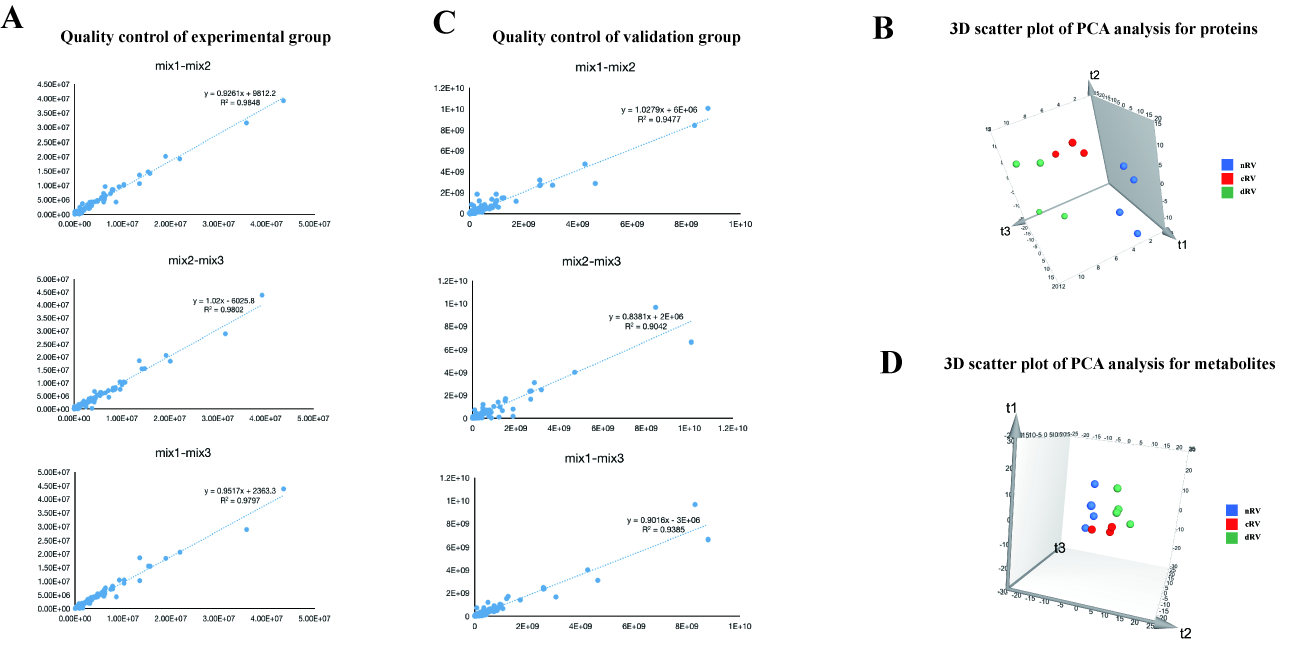

Supplement: Supplementary file 5 [file Image2.TIF]

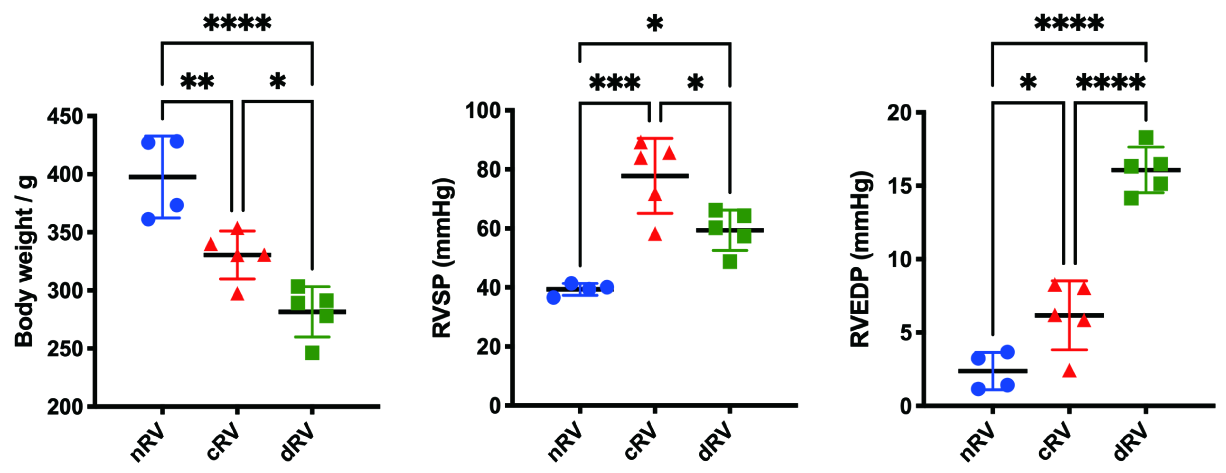

Supplement: Supplementary file 6 [file Image1.TIF]
